# Cytokine competent gut-joint migratory T Cells contribute to inflammation in the joint

**DOI:** 10.3389/fimmu.2022.932393

**Published:** 2022-09-07

**Authors:** Adam R. Lefferts, Eric Norman, David J. Claypool, Uma Kantheti, Kristine A. Kuhn

**Affiliations:** Division of Rheumatology, University of Colorado, Aurora, CO, United States

**Keywords:** spondyloarthritis (SpA), inflammatory bowel disease, T cells, cellular trafficking, inflammation, TNF, IL-17

## Abstract

Although studies have identified the presence of gut-associated cells in the enthesis of joints affected by spondylarthritis (SpA), a direct link through cellular transit between the gut and joint has yet to be formally demonstrated. Using KikGR transgenic mice to label *in situ* and track cellular trafficking from the distal colon to the joint under inflammatory conditions of both the gut and joint, we demonstrate bona-fide gut-joint trafficking of T cells from the colon epithelium, also called intraepithelial lymphocytes (IELs), to distal sites including joint enthesis, the pathogenic site of SpA. Similar to patients with SpA, colon IELs from the TNF^ΔARE/+^ mouse model of inflammatory bowel disease and SpA display heightened TNF production upon stimulation. Using *ex vivo* stimulation of photo-labeled gut-joint trafficked T cells from the popliteal lymph nodes of KikGR and KikGR TNF^ΔARE/+^ we saw that the CD4+ photo-labeled population was highly enriched for IL-17 competence in healthy as well as arthritic mice, however in the TNF^ΔARE/+^ mice these cells were additionally enriched for TNF. Using transfer of magnetically isolated IELs from TNF^+/+^ and TNF^ΔARE/+^ donors into *Rag1* ^-/-^ hosts, we confirmed that IELs can exacerbate inflammatory processes in the joint. Finally, we blocked IEL recruitment to the colon epithelium using broad spectrum antibiotics in TNF^ΔARE/+^ mice. Antibiotic-treated mice had reduced gut-joint IEL migration, contained fewer Il-17A and TNF competent CD4+ T cells, and lessened joint pathology compared to untreated littermate controls. Together these results demonstrate that pro-inflammatory colon-derived IELs can exacerbate inflammatory responses in the joint through systemic trafficking, and that interference with this process through gut-targeted approaches has therapeutic potential in SpA.

## Introduction

Spondyloarthritis (SpA) is a family of chronic inflammatory arthritides primarily targeting the joint entheses, the site of tendon or ligament insertion into bone. Enthesial inflammation can cause pain, joint damage, and loss of mobility in affected sites, leading to significant loss of quality of life (1, 2). While in recent years there have been significant advances in the treatment of SpA, headlined by biologic treatments aimed at the blockade of tumor necrosis factor (TNF) and interleukin (IL)-17 (3); however these treatments are efficacious in only ~60% of patients (4). In those patients for whom therapy is effective, ~30% of patients discontinue treatment after 12 months due to cost and treatment-associated adverse reactions (2). Therefore, additional treatment strategies are needed to reduce the burden of this disease.

Immunologically, SpA is believed to be largely caused by local overproduction of Il-17 and TNF (5). In the joint, IL-17 and TNF synergize to potentiate the production of bone-erosion promoting factors such as receptor activator of nuclear factor κβ ligand (RANKL) (5, 6), ultimately leading to joint pathology. In the human enthesis, a series of studies demonstrated that both CD4+ and CD8+ T cells isolated from the spinous process enthesis of individuals undergoing spine surgery could be stimulated to produce both IL-17A and TNF (7) into the culture supernatant, suggesting that pro-inflammatory T cell infiltrate is a critical component of disease pathology. Conversely, in bulk RNA sequencing of enthesial CD4+ and CD8+ T cells, gene expression of the T cells was consistent with a tissue repair pattern, including higher vascular endothelial growth factor (VEGF), transforming growth factor (TGF)-β, and IL-10 expression compared to their circulating counterparts (5). Thus, it appears that two types of T cell populations may exist in the enthesis: those that secrete IL-17A and/or TNF and those that are regulatory. Therefore, while the importance of T cell driven immunopathology in SpA is well supported, the mechanisms by which these cells are generated and arrive in the joint remain largely uncharacterized.

One potential clue to the pathogenesis of SpA lies in the clear clinical connection between SpA and inflammatory bowel diseases (IBD) such as Crohn’s disease and ulcerative colitis (8). As many as 10% of individuals with axial SpA (axSpA) are diagnosed with co-morbid IBD, and up to 50% of individuals with axSpA will present with sub-clinical bowel inflammation that is detectable by biopsy but is not sufficient for a diagnosis of IBD (8). This observation has led to the generation for the gut-joint hypothesis in axSpA (9–11), which proposes that inflammatory disease begins and is driven by host-microbe interactions in the gut and then spreads to distal sites, ultimately presenting as inflammatory back pain. Multiple studies have reported increases in circulating and synovial populations of Th17 polarized T cells, ILC3s, mucosa-associated invariant T cells (MAITs), and TCRγδ+ T cells (12–15), all of which are generally thought to be generated and/or activated through microbiome-mediated mechanisms at the mucosa and are capable of production of TNF and IL-17 (16), the primary mediators of disease in axSpA (3).

Work in the HLA-B27 transgenic rat model of SpA supports the idea of a gut-joint connection, as differential disease penetrance in distinct rat strains has been linked to the microbiota. Indeed, HLA-B27 itself has been shown to alter the human microbiome (17), and individuals with axSpA have distinct perturbations in their own microbial communities that have been linked to production of pro-inflammatory cytokines (18). In the SKG model, which is driven by impaired zeta-chain-associated protein kinase (ZAP)-70 function (19), the microbiome has been shown to be critical for the development of arthritic disease (20). Together these findings indicate that the gut may be an important driver of arthritis, with the microbiome perhaps accounting for some of the missing environmental risk factors for the development of SpA.

Although a substantial body of evidence supports the gut-joint hypothesis in SpA, limitations on what can be learned from observational studies in humans mean that the potential mechanisms by which such a connection might be mediated have remained largely unexamined. In our own previous work, we observed an inverse correlation between the absolute lymphocyte count in the blood and the number of intraepithelial lymphocytes (IELs, T cells within the intestinal epithelium) in colon biopsy tissue from patients with axSpA (18). This inverse relationship could be explained by the physical migration of IELs into circulation, which is in line with prior findings of increased gut-associated cell types in circulation in SpA (12, 21). We therefore hypothesized that colon IELs engage in systemic trafficking and are involved in immune responses in the joint.

To explore this hypothesis two murine models were needed, one in which systemic trafficking could be observed, and a SpA model in which the key feature of IELs in human axSpA, overproduction of TNF (18), was maintained. Previously, systemic trafficking of specific cell types to and from tissues including the gut has been shown using photoactivatable transgenic Kaede and Kikume green-red (KikGR) mice (22–24). KikGR+ mice express a photoconvertable fluorescent protein under a CMV enhancer/chicken beta-actin promoter (25). Upon exposure to 405 nm light, the KikGR protein undergoes an irreversible conformation change, which alters its fluorescent properties, and can be detected by flow cytometry and fluorescent microscopy. Using a similar transgenic approach, gut T cell trafficking to the eye could be measured in a murine model of experimental autoimmune uveitis (24).

To model IBD and SpA, we chose the TNF^ΔARE/+^ transgenic murine model given the success of TNF targeting treatments in in these diseases and our observation of increased TNF production by stimulated IELs in SpA and IBD (18). The TNF^ΔARE/+^ model of IBD and SpA is driven by a 69bp deletion in the 3’ AU-rich untranslated region of TNF mRNA, resulting in increased stability of the TNF mRNA and increased TNF protein expression in cells in which the mRNA is present (26). Similar to other models of SpA, such as the SKG and B27 transgenic rat, the TNF^ΔARE/+^ shows a dependence on the microbiota, as germ-free animals display no meaningful disease in the gut (27, 28), although arthritic disease was not assessed in these mice. CD4+ T cells are also known to be critical in the TNF^ΔARE/+^ model, as CD4+ T cells isolated from the MLN are sufficient to drive disease after transfer to *Rag1* ^-/-^ mice (29). By crossing the KikGR and TNF^ΔARE/+^ transgenic models we explored the trafficking dynamics of colon IELs and assessed their potential contribution to arthritic disease.

Here we demonstrate gut-joint trafficking of colon IELs. Using the KikGR transgenic mouse model we are able to photo-label colon IELs *in situ* in the descending colon of live mice and track subsequent migration by flow cytometry. We further observe that migratory colon IELs in the peripheral joints are highly cytokine competent and are capable of exacerbating inflammatory responses in the joint. Finally, we show that treatment with broad spectrum antibiotics reduces the number and pro-inflammatory potential of peripheral IELs, and results in a significant reduction in joint disease in the TNF^ΔARE/+^ model. These results provide an example of gut-joint trafficking and show a means by which immunity at multiple sites may be mediated in SpA.

## Materials and methods

### Mice

Recovered cryopreserved KikGR mice (Tg(CAG-KikGR)33Hadj/J, strain #013753, Jackson Laboratory), TNF^ΔARE/+^ mice (B6.129S-Tnf tm2Gkl/Jarn strain, gift from Sean Colgan), and *Rag1* ^-/-^ (B6.129S7-Rag1tm1Mom/J, strain #002216, Jackson Laboratory) were maintained as colonies at the University of Colorado Anschutz Medical Campus in specific pathogen-free housing conditions. All experimental manipulations were approved under the Institutional Animal Care and Use Committee. In all experiments age and sex matched littermate controls were used, allowing 2-1 ratios in cases where there was an odd number of animals in a litter.

Photoconversion of the distal colon was performed under anesthesia using inhaled isoflurane induced and maintained at 1- 3% using an anesthesia machine, and confirmed by the absence of withdrawal to toe pinch and skeletal muscle tone. A 7 French (2.3 mm) rigid colonoscope (Storz) was introduced into the rectum to a depth of 25mm, up to but not past the splenic flexure with direct visualization *via* attached camera. 405 nm light was shone through the scope using a 100 mW handheld laser pointer. Laser light was applied in 15 second pulses and retracted 5 mm until removed from the colon. Anesthesia was withdrawn and mice monitored until recovered.

Blockade of lymphocyte trafficking was established with the sphingosine-1-phosphate inhibitor FTY720 (Cayman) injected intraperitoneally at a dose of 1 mg/kg in phosphate-buffered saline (PBS). As a control, mice were injected with 10% dimethyl sulfoxide in PBS (Gibco). Mice were administered 4 doses, first at the time of photoconversion and subsequently every 24 hours until time of harvest three days following photoconversion.

To induce local inflammation in the hind ankle, complete Freund’s adjuvant (CFA) was injected into the hind hock according to the method of Kamala et al. (30). Briefly, mice were kept under anesthesia as above. 20ul of an emulsion of 1:1 CFA and sterile PBS was injected into the hind hock, just inside of the Achilles tendon. A sham injection was performed on the contralateral joint by simply inserting 25 gauge needle in the site. Anesthesia was withdrawn and the mice monitored for recovery.

IEL transfer from TNF^ΔARE/+^ mice and TNF^+/+^ littermate controls, harvested as described below, were transferred into *Rag1* ^-/-^ mice by tail vein injection of 100 μl volume of 5 x 10^5^ lELs in PBS, or PBS alone or *Rag1* ^-/-^ controls, using a 25-gauge needle. Blood was collected by submandibular bleed two times a week for the quantification of circulating T cells.

Antibiotic treatment was administered ad libitum in the drinking water from 4 -16 weeks of age. The antibiotic cocktail contained 1 mg/ml each ampicillin (Research Products International, RPI) and neomycin (RPI), and 0.5 mg/ml each metronidazole (RPI) and vancomycin (Alfa Aesar). To encourage consumption 20 mg/ml sugar-sweetened grape-flavored Kool-Aid (Kraft) was added. Control mice were treated with Kool-Aid, but no antibiotics, added to their drinking water.

Quantification of the gene for 16S ribosomal RNA was carried out using the QIAamp PowerFecal Pro DNA Kit (Qiagen). The 16S rRNA gene was amplified using CYBRFast master mix (Tonbo) with 5’-cccgatggtataatcagac-3’ forward and 5’-cacaactgacttaactgtcc-3’ reverse primers on an ABI 7500 real-time PCR system.

### Flow cytometry

Flow cytometry was performed on a five laser Cytek Aurora, with some control experiments performed using a five laser BD LSR-20 Fortessa. Post-harvest tissue samples were handled in black walled conical tubes (Corning) to prevent incidental light from causing non-specific photoconversion of the KikGR protein. Achilles enthesis were harvested and incubated for 20 minutes at 37C in RPMI 1640 (Gibco) supplemented with 10% fetal bovine serum (FBS) (Sigma), 2.5 μl/ml beta-mercaptoethanol (Gibco), 0.05 M 4-(2-hydroxyethyl)-1-piperazineethanesulfonic acid (HEPES), and 0.54 mg/ml type 4 collagenase (Worthington). About 8mm of the Achilles tendon starting from the enthesis was removed. The tendon was cut free and any attachments to the gastrocnemius scraped away with forceps. About 1-2 mm of plantaris enthesis proximal to the ankle was also included. Enthesial tissue was taken from both hind paws in this manner. Tissues were then passed through a 70 μm cell strainer (VWR) and stained. Lymph nodes were homogenized with the plunger of a syringed and passed through a 70 μm cell strainer and stained. Colon tissue was harvested and flushed with PBS (Gibco) before being finely minced and placed in a 50 ml conical tube (Falcon) with 10 ml of PBS supplemented with 1 mM ethylenediaminetetraacetic acid (EDTA) and 7.5 mM HEPES. Tissue was shaken at max speed in a tabletop vortex for 10 minutes to disassociate the epithelial fraction. The epithelial fraction was passed through a 70um cell strainer and proceeded to staining. Intact tissue as taken from the strainer and re-minced until no visible pieces of tissue remained. Tissue was incubated with shaking in at 37C in 2ml RPMI 1640 (Gibco) supplemented with 10% FBS (Sigma), 2.5 μl/ml beta-mercaptoethanol (Gibco), 0.05 M HEPES and 0.54 mg/ml type 4 collagenase (Worthington). Cells were washed to remove collagenase and passed through a 70um cell strainer (VWR) before proceeding to staining. Staining took place for 30 minutes at 4C in PBS supplemented with 10% FBS (Sigma) with the indicated antibodies at 1 part in 100. Cells were then washed in PBS, pelleted at 500g for ten minutes and fixed for 20 minutes using the FoxP3/Transcription Factor Staining Buffer Kit (Tonbo). If intracellular staining was performed, cells were additionally stained for 30 minutes at 4C with the indicated antibody and washed in PBS and pelleted before use. Antibodies, clones, fluorophore, and sources are listed in **Supplementary Table 1**. Data was analyzed using FlowJo (v10) and statistical analysis performed in GraphPad Prism v9.3.0.

### IEL isolation and reconstitution

Colon tissue was harvested and flushed with PBS (Gibco) before being finely minced and placed in a 50 ml conical tube (Falcon) with 10 ml of PBS supplemented with 1 mM EDTA and 7.5 mM HEPES. Tissue was shaken at max speed in a tabletop vortex for 10 minutes to disassociate the epithelial fraction. The epithelial fraction was passed through a 70um cell strainer (VWR) before proceeding to magnetic enrichment. IELs were purified using a Easysep negative selection magnetic enrichment kit (STEMCELL), supplemented with 1ul/sample biotin conjugated anti-EPCAM (clone G8.8). Isolated cells were introduced to *Rag1* ^-/-^ hosts by tail vein injection. Homeostatic T cell proliferation in recipient animals was monitored weekly by cheek bleed and flow cytometry until circulating CD3+ lymphocyte levels were stable (10 weeks).

### *Ex vivo* cell stimulation

Popliteal lymph nodes were harvested and enriched for T cells using the EasySep magnetic negative selection kit (STEMCELL). Cell suspensions were placed in RPMI 1640 completed with 10% FBS (Sigma) and stimulated with anti-CD3/CD28 conjugated Dynabeads (Gibco) in the presence of protein transport inhibitor cocktail (eBioscience). Cells were stimulated for 4 hours before harvest and intracellular cytokine staining.

### Histology

Colon and ileal tissue was harvested and pined flat before fixation for 24 hours with 4% paraformaldehyde. Fixed tissue was rolled prior to paraffin embedding, and 5 μm sections stained with hematoxylin and eosin. Ankle and knee tissue was decalcified using treatment with formic acid for 7 days. Tissues were washed with 70% ethanol before being blocked in paraffin, sectioned at 5 μm, and stained with hematoxylin and eosin. Scoring of all histology was performed blinded to the experimental condition. Colon and ileal pathology were determined through direct measurements taken from in-frame regions of taken from the whole length of the colon and the whole length of the ileum in Photoshop (Adobe). At minimum, 5 images were assessed from each mouse with at least 5 measurements taken per image. Ankles and knees were scored from 0-4 (0=none; 1=minimal; 2= mild; 3=moderate; 4=severe) on each of the following metrics, and results summed to generate a histopathology score: inflammatory infiltrate, synovio-enthesitis, cartilage loss, and bone destruction.

## Results

### Colon IELs engage in constitutive gut-joint trafficking

**Figure 1:**
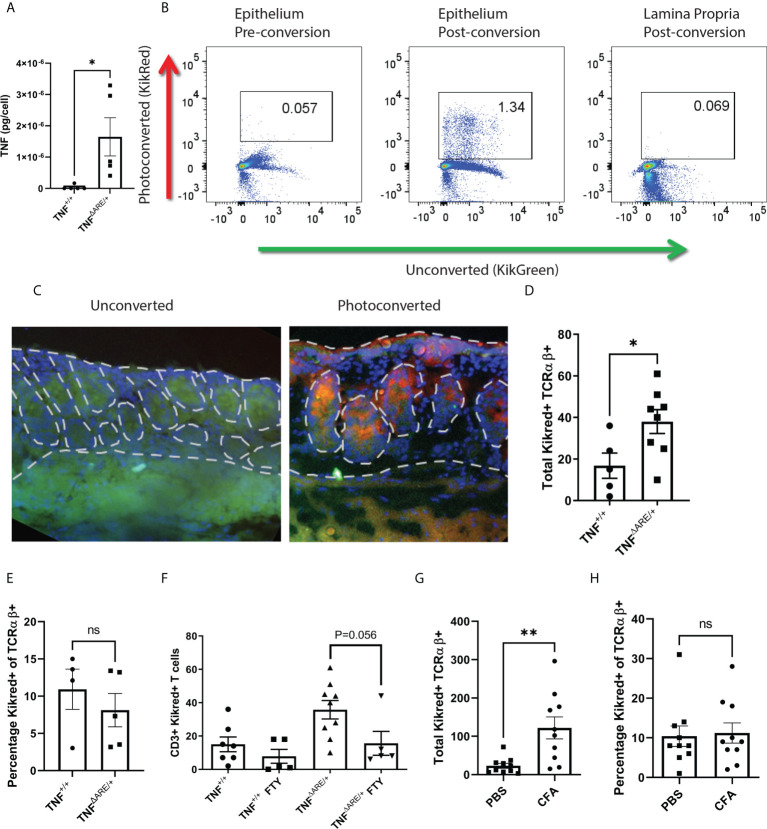
IELs engage in constitutive gut-joint trafficking. IELs were isolated from the colon epithelia of 8-10 week old male and female TNF^ΔARE/+^ mice (n=5) and TNF^+/+^ littermate controls (N=5). **(A)** IELs were stimulated with PMA and Ionomycin overnight, the supernatant was assayed for TNF by ELISA and per cell TNF production calculated. Each symbol represents an individual mouse with bars as mean ± SEM. *P<0.05 by Student’s t-test. **(B)** KikGreen versus KikRed protein signal was compared by flow cytometry before and after photoconversion in the epithelium and lamina propria. **(C)** Representative immunofluorescence at 400X magnification is shown of colon tissue harvested from a KikGR+ mouse that had just undergone photoconversion and from an unconverted control. **(D, E)** 72 hours post photo-conversion the Achilles enthesis was harvested from TNF^ΔARE/+^ (n=8) and TNF^+/+^ littermate controls (n=5) and assayed for the presence of photoconverted KikRed+ CD3+ TCRαβ+ T cells by flow cytometry. **(D)** Absolute number and **(E)** percentage is shown for each mouse (symbols) with mean ± SEM (bars). **(F)** 8-10 week old male and female KikGR+ mice received a photoconversion of the distal colon and daily i.p. injections of 1mg/kg FTY720 or 10% DMSO vehicle control. Absolute number of KikRed+ CD3+ TCRαβ+ T cells were measured by flow cytometry. **(G, H)** KikGR^+^ mice (n=10) received an injection of 20mg CFA in the hind hock and a injection of PBS in the contralateral hock simultaneously to having undergone photoconversion of the distal colon. 72 hours post injection, each Achilles enthesis was harvested and **(G)** total and **(H)** percentage of KikRed+ CD3+ TCRαβ+ T cells were enumerated by flow cytometry. Data are shown as individual mice across three separate experiments with bars as mean ± SEM. *P<0.05; **P<0.01 , and ns, non-significant as determined by Student’s t-test, and paired Student’s T test for **(G, H)**.

Previously, we demonstrated that IELs from patients with either axSpA or Crohn’s disease are enriched for TNF production compared to controls (18). We confirmed that, similar to human axSpA, IELs isolated from TNF^ΔARE/+^ mice have increased TNF production upon stimulation relative to those isolated from TNF^+/+^ littermate controls (**Figure 1A**). Next, because or prior work in human axSpA demonstrated an inverse correlation between the number of colon IELs and circulating lymphocytes (18), we tested the hypothesis that colon IELs engaged in systemic trafficking by crossing TNF^ΔARE/+^ mice with the transgenic KikGR model. Utilizing timed exposures of the distal colon during colonoscopy of 15 seconds per 5 mm tissue, the epithelial fraction can be photo-labeled without significant exposure into the lamina propria as confirmed by both flow cytometry (**Figure 1B**) and histologically (**Figure 1C**). Using this model, we observed the presence of KikRed+ photo-labeled colon-derived IELs in the Achilles enthesis, the site of pathogenic inflammation in SpA (3), peaking 72 hours after photoconversion in arthritic TNF^ΔARE/+^ mice as well as TNF^+/+^ littermate controls (**Figure 1D**; **Supplementary Figure 1A**). The majority of trafficked KikRed+ IELs in the enthesis were verified to be within tissue rather than the vasculature of the tissue, as injection with anti-CD3 APC just prior to euthanasia to identify intravascular T cells represented only ~20% of the KikRed+ TCRβ+ CD4+ cells (**Supplementary Figure 1B**). We also observed trafficking to other tissue sites, including the lung, liver, and skin, but was significantly reduced in magnitude and more commonly intravascular compared to what was observed in the joint (**Supplementary Figure 1C**).

Our work with colon IELs has highlighted an important difference from small intestinal IELs, in that those in the colon are αβ+ T cells that are equally distributed among CD4+, CD8+, and CD4-CD8- subsets (31). Therefore, we focused on TCRαβ+ T cells. While the total number of photo-labeled KikRed+ CD3+ TCRαβ+ T cells was significantly increased in the arthritic TNF^ΔARE/+^ mice (**Figure 1D**), there was no difference in the percentage of photo-labeled cells between groups (**Figure 1E**), suggesting that trafficking IELs are a stable component of circulating T cell surveillance of tissues. We additionally confirmed that these cells were indeed engaged in systemic trafficking using the inhibitor of systemic T cell trafficking FTY720, which disrupts the sphingosine-1-phosphate (S1P)- sphingosine-1-phosphate receptor (S1Pr) signaling axis and results in sequestration of T cells in the lymph (32). FTY720 1 mg/kg was administered intraperitoneally to TNF^ΔARE/+^ and TNF^+/+^ mice at the time of photoconversion and daily for 72 hours, after which the Achilles enthesis was harvested and analyzed for KikRed+ colon IEL trafficking. We observed a significant reduction in trafficked KikRed+ CD3+ TCRαβ+ T cells in the enthesis of the TNF^ΔARE/+^ mice with a trend towards but not significant reduction in TNF^+/+^ mice, likely due to the small number of T cells observed in the enthesis at steady state (**Figure 1F**).

Given that the TNF^ΔARE/+^ model is of chronic systemic inflammation driven by dysregulated TNF expression, we also evaluated gut-trafficked T cells in an acute model of joint inflammation. Hock-injection with complete Freund’s adjuvant (CFA) in KikGR mice produces rapid inflammation characterized by neutrophil infiltration, periosteal inflammation, synovitis and enthesitis (**Supplementary Figure 2**). We performed photoconversion of the colon at the time of hock injection, and again observed significantly increased gut-derived KikRed+ CD3+ TCRαβ+ T cells in the Achilles enthesis after 72 hours (**Figure 1G**). Similar to our findings in TNF^ΔARE/+^ mice, the proportion of gut-joint trafficked T cells did not differ between the CFA-injected and PBS-injected Achilles entheses (**Figure 1H**). These results establish the direct trafficking of gut-derived migratory IELs in peripheral tissues including the joint enthesis and imply that they are a component of homeostatic immune surveillance.

### Colon IELs maintain cytokine competence following systemic trafficking

**Figure 2:**
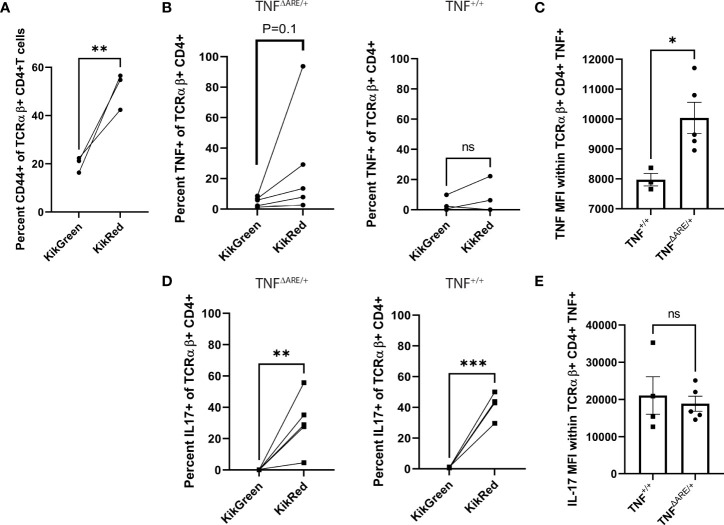
IELs maintain elevated cytokine competence following systemic trafficking. 8-10 week old male and female KikGR^+^ TNF^+/+^ (N=4) and KikGR^+^ TNF^ΔARE/+^ mice (N=5) underwent photoconversion of the distal colon. Popliteal lymph nodes (PLNs) were harvested 72 hours later. T cells were purified by magnetic negative selection and stimulated with bead-bound anti-CD3 and anti-CD28 in the presence of protein transport inhibitor for 4 hours. **(A)** The percentage of CD44+ KikGreen+ TCRαβ+ CD4+ versus CD44+ KikRed+ TCRαβ+ CD4+ T cells was evaluated by flow cytometry in TNF^+/+^ mice. **(B)** The percentage of KikGreen+ versus KikRed+ TCRαβ+ CD4+ T cells that were positive for TNF was evaluated by flow cytometry in TNF^ΔARE/+^ (Left) and TNF^+/+^ (Right) mice. **(C)** Mean fluorescence intensity (MFI, arbitrary units) of TNF was determined within the TNF+ TCRαβ+ CD4+ population. **(D)** The percentage of KikGreen+ versus KikRed+ TCRαβ+ CD4+ T cells that were positive for IL-17A was evaluated by flow cytometry in TNF^ΔARE/+^ (Left) and TNF^+/+^ (Right) mice. **(E)** Mean fluorescence intensity (MFI, arbitrary units) of IL-17A was determined within the IL-17A+ TCRαβ+ CD4+ population. Data are shown as individual mice (symbols) across two separate experiments with bars as mean ± SEM. *P<0.05; **P<0.01; ***P<0.0001 as determined by Student’s T-test for **(B, D)** and, and paired Student’s T test for **(A, C)** ns, not significant.

In order to explore the potential role of colon-derived IELs that have trafficked to the joint, we examined cytokine production following *ex vivo* stimulation. Due to the small cell numbers in the Achilles enthesis, we examined the gut-trafficked KikRed+ T cells from the draining popliteal lymph node (PLN) as the most meaningful proxy for the lymphocytes that would have the capability the enter the enthesis. Initially, we observed an increase in CD44+ cells in the KikRed+ fraction (**Figure 2A**), indicative of antigen exposure (33). Seventy-two hours following photoconversions of the distal colon of both un-diseased TNF^+/+^ and arthritic 12-week-old TNF^ΔARE/+^ mice, CD4+ T cells from the PLNs were harvested using magnetic negative selection and stimulated with bead-bound anti-CD3 and anti-CD28 for four hours in the presence of protein transport blockade. By flow cytometry, we compared cytokine production by CD4+ T cells in the unconverted KikGreen+ fraction versus the photo-converted, gut-derived KikRed+ fraction (representative gating shown in **Supplementary Figure 3A**). We observed a slight but non-significant increase in TNF-producing KikRed+ CD4+ T cells versus KikGreen+ CD4+ T cells from TNF^ΔARE/+^ mice, with no difference between TNF-producing KikRed+ versus KikGreen+ CD4 T cells in TNF^+/+^ mice (**Figure 2B**). The TNF+ KikRed+ CD4+ T cells isolated from TNF^ΔARE/+^ mice produced significantly more TNF compared to those cells from TNF^+/+^ mice as measured by mean fluorescence intensity (MFI) (**Figure 2C**), suggestive of increased TNF expression upon stimulation and paralleling the previous finding of increased TNF production by colon IELs (**Figure 1A**). Together these data indicate that the pro-inflammatory capacity of colon IELs in TNF^ΔARE/+^ mice was maintained as these cells trafficked systemically.

**Figure 3:**
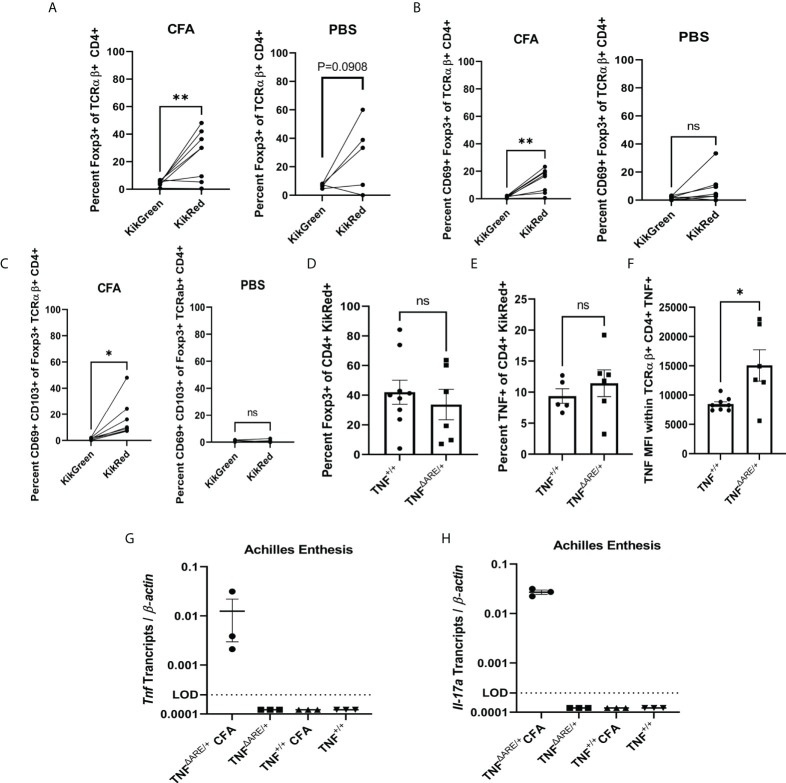
Injury induces influx of gut-derived regulatory T cells. 8-10 week old male and female KikGR^+^ (n=5) mice received an injection CFA in the hind hock and PBS in the contralateral hock simultaneously to having undergone photoconversion of the distal colon. 72 hours post injection each Achilles enthesis was harvested and **(A)** Foxp3+ TCRαβ+ CD4+ T cells, **(B)** CD69+ Foxp3+ TCRαβ+ CD4+ T cells and **(C)** CD69+ CD103+ TCRαβ+ CD4+ T cells were enumerated in the CFA-injected hock (left) and PBS-injected hock (right) by flow cytometry. **(D-F)** 8-10 week old male and female KikGR^+^ TNF^ΔARE/+^ (n=6) and TNF^+/+^ littermate controls (n=9) received an injection of CFA in the hind hock and an injection of PBS in the contralateral hock simultaneously to having undergone photoconversion of the distal colon. 72 hours post injection each Achilles enthesis was harvested and **(D)** KikRed+ Foxp3+ TCRαβ+ CD4+ T cells and **(E)** KikRed+ TNF+ TCRαβ+ CD4+ T cells were enumerated by flow cytometry. **(F)** Mean fluorescence intensity (arbitrary units) of TNF was determined within the TNF+ TCRαβ+ CD4+ population. **(G, H)** 8-10 week old male and female TNF^ΔARE/+^ (n=3) and TNF^+/+^ littermate controls (n=3) received an injection of CFA in the hind hock and an injection of PBS in the contralateral hock. After 72 hours each Achilles tendon was removed and RNA isolated. RT-qPCR was performed to determine the levels of **(G)** *Tnf* and **(H)** *Il-17a* transcript relative to *β-actin*. Data are shown as individual mice (symbols) with bars as mean ± SEM. Significance was determined by paired Student’s T test for **(A–C)** and unpaired Student’s T-test for **(D–H)**. *,P<0.05 and **,P<0.01; ns, not significant.

Given the role of IL-17 in the pathophysiology of SpA (21) and prior descriptions of IL-17 producing cells in the synovium of individuals with SpA (34), we additionally evaluated for the production of this cytokine. IL-17 producing T cells were significantly enriched within the KikRed+ CD4+ T cells versus the KikGreen+ fraction of both TNF^ΔARE/+^ and TNF^+/+^ mice (**Figure 2D**), with no significant difference in MFI between healthy and arthritic animals (**Figure 2E**). These data indicate that the high TNF production capacity of IELs in the TNF^ΔARE/+^ is maintained following egress from the gut and trafficking to distal sites, and demonstrate a significant enrichment for IL-17 competence within the gut-joint migratory T cell subset in both healthy and diseased mice.

### Injury also induces influx of gut-derived regulatory T cells

In the human enthesis, both CD4+ and CD8+ T cells could be stimulated to produce both IL-17A and TNF while bulk gene expression of the T cells was consistent with a tissue repair pattern including higher VEGF, TGF-β, and IL-10 expression compared to their circulating counterparts (7). Thus, it appears that two types of T cell populations may exist in the enthesis: those that secrete IL-17A and/or TNF and those that are regulatory. Therefore, we evaluated whether the gut-trafficked KikRed+ IELs in the joint had a regulatory subpopulation. First, we utilized our hock-injection model, simultaneously performing photoconversions of the distal colon injection of 20mg CFA into the hind hock as before. After 72 hours, we interrogated T cells in the PLNs by flow (representative gating shown in **Supplementary Figure 3B**). CD4+ FoxP3+ T cells were enriched within the KikRed+ but not KikGreen+ populations following CFA injection (**Figure 3A**), which was not mirrored in the PBS-injected side. The gut-derived KikRed+ CD4+ FoxP3+ regulatory T cells were also enriched for the activation marker CD69 in the PLNs from the CFA but not PBS-injected hocks (**Figure 3B**), and within that population also displayed increased CD103+ expression which could be indicative of alterations to trafficking (**Figure 3C**). CD69 enrichment was not mirrored in the general KikRed+ CD4+ T cell population (**Supplementary Figure 4A**), indicating that inflammatory injury results in specific activation of gut-derived regulatory T cells.

To investigate the hypothesis that joint pathology in TNF^ΔARE/+^ mice may be at least partially due to a deficiency in the recruitment of gut-derived T regulatory cells, we repeated the simultaneous photoconversions and hock-injections as above in TNF^ΔARE/+^ mice versus TNF^+/+^ littermate controls. We observed similar magnitudes of enrichment for KikRed+ CD4+ FoxP3+ regulatory T cells following injury in both TNF^ΔARE/+^ and TNF^+/+^ mice (**Figure 3D**) as well as total CD4+ FoxP3+ regulatory T cells (**Supplementary Figure 4B**). Since we had observed a slight increase in TNF competent cells in the KikRed+ gut derived population in the PLNs of TNF^ΔARE/+^ mice, we also checked if there was an enrichment of TNF competent cells following hock-injection. We observed similar numbers of TNF+ CD4+ T cells in the injured joint in TNF^ΔARE/+^ and TNF^+/+^ mice (**Figure 3E**); however, similar to our findings at steady state, following injury we observed an increase in the MFI of TNF within TNF+ CD4+ T cells in TNF^ΔARE/+^ mice (**Figure 3F**). We also observed both *Tnf* and *Il17a* mRNA in the Achilles enthesis of TNF^ΔARE/+^ mice following induced injury, which were undetectable in the uninjured Achilles from TNF^ΔARE/+^ or either CFA- or PBS-injected Achilles in TNF^+/+^ mice (**Figures 3G, H**). These findings indicate that following injury there is an influx of gut-derived regulatory cells into the joint, and that this process is present in both healthy and arthritic animals. However, in the arthritic TNF^ΔARE/+^ mice, there is also increased local TNF and IL-17A production which may be an important factor that skews the response from pro-resolving to pro-inflammatory.

**Figure 4:**
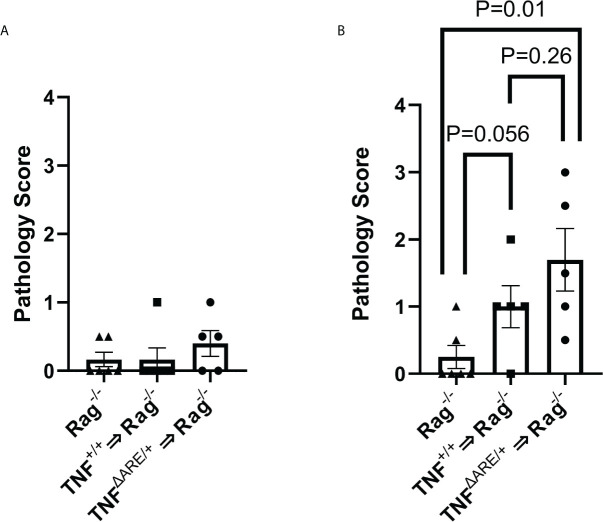
IELs from TNF^ΔARE^ exacerbate induced joint inflammation. IELs from TNF^+/+^ and TNF^ΔARE/+^ mice were harvested from the colon epithelium, purified by magnetic negative selection and transferred into *Rag1* ^-/-^ recipients by tail vein injection; PBS injection was used as a non-transfer control. Following 10 weeks of homeostatic proliferation, recipients of IELs derived from TNF^+/+^ mice (n=5), TNF^ΔARE/+^ mice (n=5), and *Rag1* ^-/-^ PBS controls (n=6) received an injection of 20mg CFA in one hind hock and PBS in the other. Mice were euthanized 5 days following hock injections and ankles evaluated histologically. Ankles were scored from 0-4 (0=none; 1=minimal; 2= mild; 3=moderate; 4=severe) on each of the following metrics, and results summed to generate a histopathology score: synovio-enthesitis, cartilage loss, and bone destruction in the **(A)** PBS-injected hock and **(B)** CFA-injected hock. Data are shown as individual mice (symbols) with bars as mean ± SEM. P values shown were determined by Student’s T test; ns, not significant.

### Colon IELs exacerbate inflammation in the joint

The confirmation of systemic trafficking and elevated cytokine production within the KikRed+ trafficked IELs in the PLNs led us to investigate if these cells could directly contribute to or induce pathology in the joint. Because colon IELs are a heterogeneous population composed of multiple T cell lineages and without specific ontogenetic markers, deletion of these cells by genetic manipulation is not possible. Therefore, we transferred 5 x 10^5^ lELs, which were harvested and magnetically enriched from TNF^ΔARE/+^ and TNF^+/+^ colons (**Supplementary Figures 5A-C**) into *Rag1* ^-/-^ mice, which lack an adaptive immune system (35), and allowed for homeostatic proliferation over 10 weeks. As an injection control *Rag1* ^-/-^ mice were given an equal volume of PBS and aged alongside the mice with IELs transferred. Eight weeks following IEL transfer, the right hock was injected with 20mg of CFA to induce local inflammation, testing the role of IELs in contributing to joint inflammation. The left hock was PBS-injected to determine if IEL transfer alone was sufficient to drive joint pathology. After 5 days, mice were euthanized and the hind ankles evaluated by a blinded observer for histopathology including inflammatory infiltrate, synovio-enthesitis, cartilage loss and bone destruction. In the PBS-injected ankles we observed no increase in pathology relative to the *Rag1* ^-/-^ controls given PBS injections (**Figure 4A**) indicating that IELs alone are not sufficient to drive joint pathology. Following CFA injection, we observed an increase in joint pathology in the mice that received colon IELs from TNF^+/+^ mice, and a more significant increase in those that received colon IELs from TNF^ΔARE/+^ mice (**Figure 4B**; **Supplementary Figures 5D-F**). These results indicate that colon IELs are capable of exacerbating an inflammatory insult in the joint, with those predisposed towards a more inflammatory profile causing worse pathology, but alone are not sufficient to drive disease.

### Antibiotics ameliorate arthritis as well as intestinal inflammation in TNF^ΔARE/+^ mice

**Figure 5:**
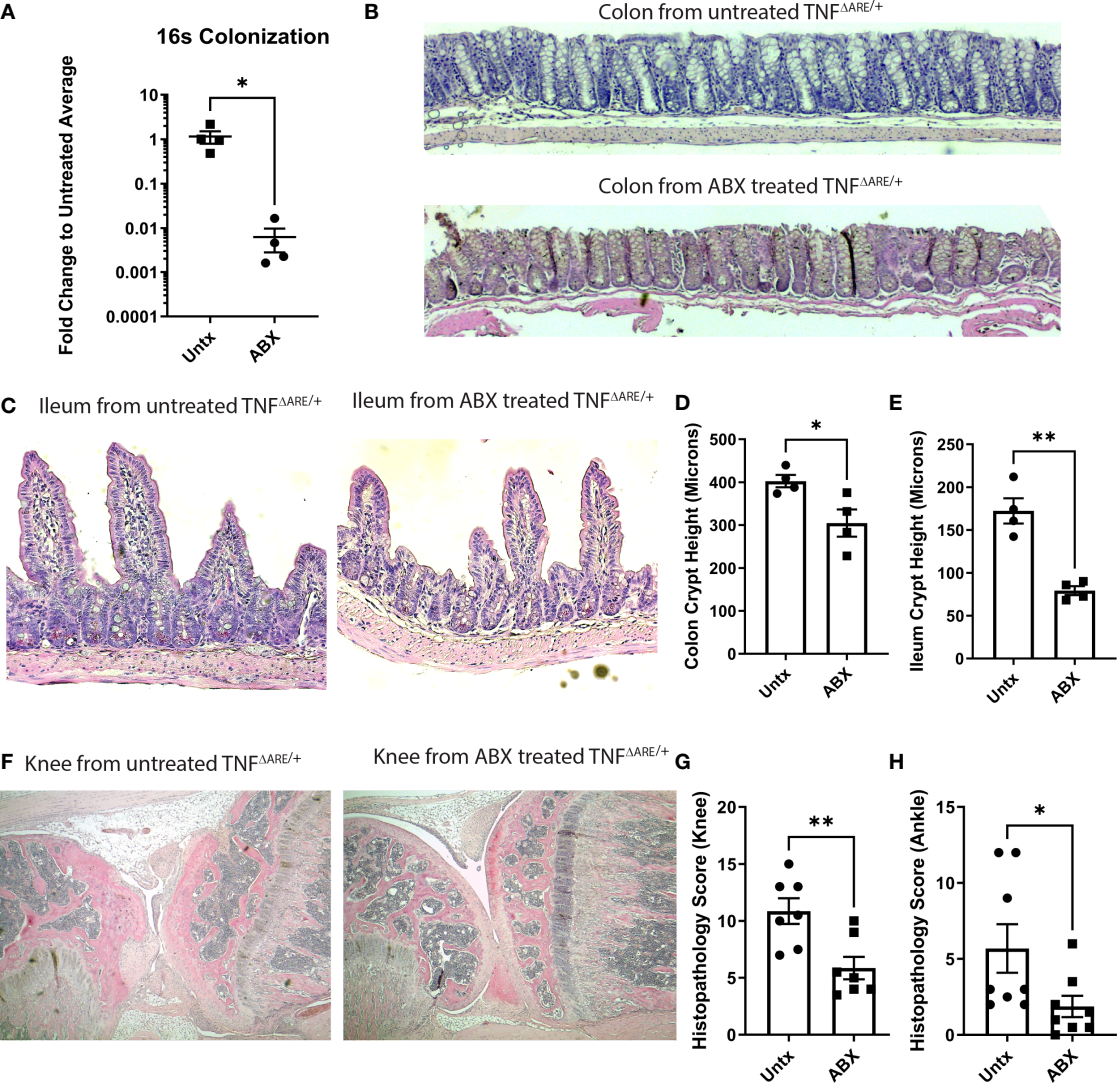
Antibiotics ameliorate disease in TNF^ΔARE/+^ mice. Male and female KikGR+ TNF^ΔARE/+^ mice were given either broad spectrum antibiotics, consisting of ampicillin, neomycin, metronidazole, and vancomycin in 10% grape Kool-Aid (n=7) or Kool-Aid alone as vehicle (n=7) ad libitum in the drinking water from 4-16 weeks of age. **(A)** Depletion of bacteria was assessed by 16S rRNA gene qPCR. At 16 weeks mice were euthanized and the colon, terminal ileum, knees, and ankles were harvested and processed for histology. **(B, C)** Representative histology of the **(B)** colon and **(C)** ileum from a control and antibiotic (ABX) treated TNF^ΔARE/+^ mouse is shown at 100X. Histology of the **(D)** colon and **(E)** ileum was evaluated for crypt height assessed in 5 fields viewed at 100X magnification. The mean length in microns for each individual mouse is represented as a symbol while the bars are the mean ± SEM for the group. **(F)** Representative histology of the knee from a control (left) and ABX treated (right) mouse is shown at 40X magnification. Histopathology of the **(G)** ankles and **(H)** knees were scored by a blinded reviewer from 0-4 (0=none; 1=minimal; 2= mild; 3=moderate; 4=severe) on each of the following metrics, and results summed to generate a histopathology score: inflammatory infiltrate, synovio-enthesitis, cartilage loss, and bone destruction. Data are shown as individual mice (symbols) with bars as mean ± SEM. *P<0.05; **P<0.01 as determined by Student’s T test.

Given our results that gut-primed IELs in the joint are capable of inflammatory cytokine production and exacerbation of inflammatory responses in the joint, we wanted to determine if disease pathology in the joint could be altered with an intervention targeting the gut. Previous work has shown that treatment with broad spectrum antibiotics largely prevents co-localization of T cells and the colon epithelium (31). Furthermore, gut pathology in TNF^ΔARE/+^ mice is dependent upon the presence of the microbiome as germ free derived TNF^ΔARE/+^ mice do not develop ileitis (27, 28). Therefore, we queried if depletion of the microbiome with broad-spectrum antibiotics (**Figure 5A**), which would both block IEL recruitment and limit disease in the gut, would serve to ameliorate arthritis. TNF^ΔARE/+^ mice were treated from 4 weeks of age, before the development of any pathology (26), until 16 weeks of age with antibiotics or vehicle in their drinking water. As expected, antibiotic treatment significantly reduced pathology in the colon and terminal ileum (**Figures 5B-E**). Additionally, antibiotic treatment compared to vehicle significantly reduced pathology in the knees and ankles (**Figures 5F-H**). These results demonstrate the importance of the microbiota in arthritis pathogenesis as well as intestinal disease and suggest potential for therapeutic interventions targeting the gut in SpA.

### Treating TNF^ΔARE/+^ mice with antibiotics reduces gut-joint trafficking and cytokine competence in CD4+ T cells

**Figure 6:**
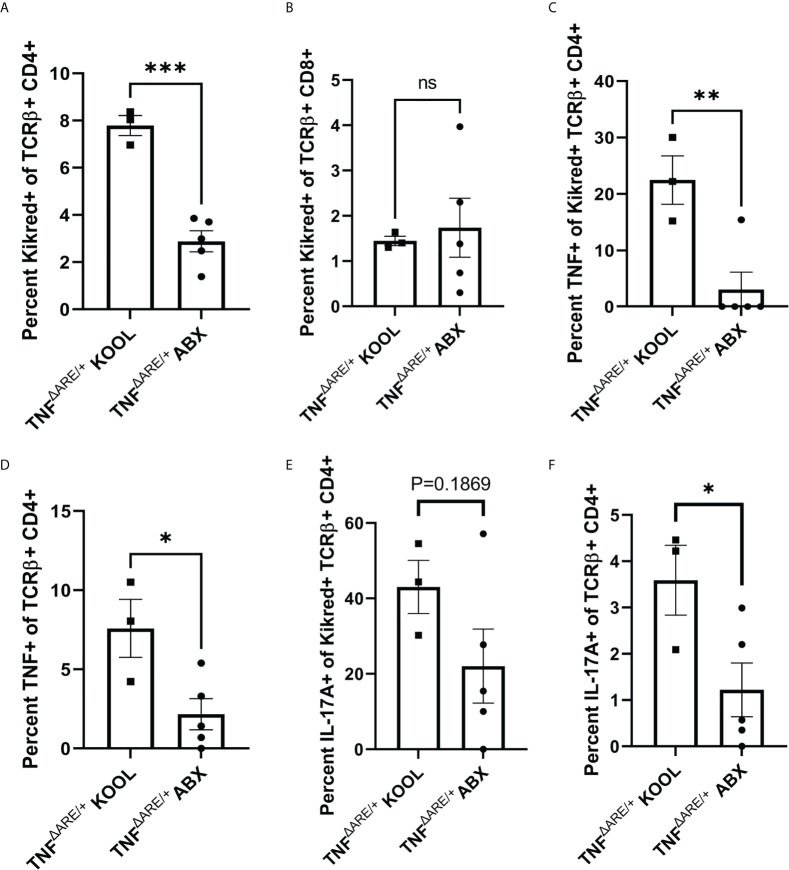
Treating TNF^ΔARE/+^ mice with antibiotics reduces gut-joint trafficking and cytokine competence in CD4+ T cells. Male and female KikGR+ TNF^ΔARE/+^ mice were given either broad spectrum antibiotics, consisting of ampicillin, neomycin, metronidazole, and vancomycin in 10% grape Kool-Aid (n=5) or Kool-Aid alone as vehicle control (n=3) ad libitum in the drinking water from 4-16 weeks of age. At 16 weeks old the mice underwent photoconversion of the distal colon and the PLNs harvested 72 hours later. T cells were purified by magnetic negative selection and stimulated with bead-bound anti-CD3 and anti-CD28 in the presence of protein transport inhibitor for 4 hours. **(A)** The percentage of KikRed+ cells within the TCRαβ+ CD4+ population and **(B)** TCRαβ+ CD8+ population was assessed by flow cytometry. The percentage of TNF+ cells within the KikRed+ TCRαβ+ CD4+ population **(C)** as well as the global TCRαβ+ CD4+ **(D)** was determined by flow cytometry. The percentage of IL-17A+ cells within the KikRed+ TCRαβ+ CD4+ population **(E)** as well as the global TCRαβ+ CD4+ **(F)** was also determined by flow cytometry. Data are shown as individual animals with bars as mean ± SEM. *P<0.05; **P<0.01; ***P<0.001; as determined by Student’s T test.

Next, to understand how antibiotic treatment affected the gut joint migratory IELs, we repeated antibiotic treatment in KikGR+ TNF^ΔARE/+^ mice from ages 4 to 16 weeks, and additionally performed a photoconversion of the distal colon at 72 hours prior to tissue harvest. We isolated CD4+ T cells from the PLN and performed stimulation and intra-cellular cytokine staining to assess T cell function in both the labeled KikRed+ fraction and the bulk KikGreen fraction. In the antibiotic treated mice compared to vehicle treatment, there was a significant reduction in the number of gut-derived KikRed+ CD4+ T cells in the PLN (**Figure 6A**) indicating that antibiotic treatment indeed reduced CD4+ T cell-epithelium colocalization resulting in less gut-joint trafficking. We did not observe a reduction in the presence of gut-derived KikRed+ CD8+ T cells following antibiotic treatment (**Figure 6B**). Within the remaining gut-trafficked cells, we also observed significant reductions in TNF competence (**Figure 6C**) and a non-significant trend towards decreased IL-17A competence (**Figure 6E**), suggesting antibiotic treatment altered those interactions with the epithelium that are important for the generation of cytokine competence within the IEL population. We also observed reductions in Il-17a and TNF competence within the unlabeled KikGreen+ population (**Figures 6D, F**), showing that sustained reduction in the number of gut derived cells that entered the periphery subsequently reduced the total number of cytokine competent cells in the animal. Together these results show that gut-joint trafficking of IELs is a constitutive process in which T cells gain effector function in the gut and contribute to inflammatory processes at distal sites.

## Discussion

Here we directly demonstrate trafficking of colon IELs to the Achilles enthesis and draining PLNs where they exhibit functional capacity to exacerbate inflammatory responses. Our results exemplify a means by which events at the gut may drive the chronic joint inflammation observed in SpA and point to a general mechanism by which conditions at the gut may influence systemic immunity. Prior work in the similar photo-convertible Kaede transgenic mouse had shown trafficking of multiple cell types including lymphocytes from the gut into lymph nodes throughout the animal (22), and into the eye (24). Our work extends this finding, showing migration from the gut not merely into important anatomic structures but also the capability of these gut-derived cells to influence local immune responses, and that interventions targeting the gut can impact disease outcomes in these sites.

We do note a couple caveats to the interpretation of our data. First, the finding of gut-derived cells in peripheral tissue as well as lymphoid structures, coupled with prior findings of trafficking from lymphoid structures to other tissue sites (22), implies the possibility that any given immune cell may have visited the intestinal epithelium or any other disease-associated tissue at some point in its developmental path. Thus, crosstalk between sites is not unique to the gut-joint axis, and our findings merely represent an example of a broader immunological principle still under investigation. Second, in this model it is unclear whether trafficking IELs represent a unique subset within the general mature T cell population due to their programming at the epithelium, or if our finding of enhanced cytokine production by IELs in the periphery represents an enrichment of mature T cell phenotypes within that population. Regardless, it is clear that these cells are potentially important producers of cytokines in the periphery.

Currently, little is known about the specific means by which interactions at the epithelium influence colon IEL function. Work using transgenic antigen expression has shown that antigen presentation by colon epithelial cells has the capability to drive Treg expansion (36). Conversely, colon epithelial cells isolated from human IBD patients have displayed the capability to drive pro-inflammatory T cell function (37). These findings suggest that situationally, the interaction between IELs and the epithelium has the capability to drive responses in either a pro- or anti-inflammatory direction; however the ultimate determinants of IEL function are yet to be determined. As a migratory subset of T cells, IELs may also be involved in more classical interactions with antigen-presenting cells in the lymph nodes. This allows for the idea that the highly functional subset of cells labeled at the gut and observed in the periphery did not gain effector function at the epithelium but simply represented a highly migratory subset of a larger pool of circulating T cells.

Our results suggest that, regardless of the specific mechanisms of their activation, trafficking of T cells to and from the colon epithelium and to distal sites including the joint is a homeostatic process that requires no specific induction. We found that ~10% of T cells in the enthesis had been present in the colon epithelium 72 hours prior. This percentage was consistent irrespective of whether the joint was healthy, chronically inflamed, or experiencing an acute inflammatory insult. These results suggest that immune surveillance of the joint occurs at homeostasis, likely in response to some factor produced in the joint itself. Isolated human chondrocytes have been shown to produce the T cell chemoattractant (38) sphingosine-1-phosphate (S1P) upon mechanical stretch (39), suggesting that mechanical loading of the joint may induce T cell recruitment into the enthesial microenvironment. Certainly reduction of the mechanical load on the joint can ameliorate arthritic disease in mice (5), but the effects of exercise in humans in less clear (40). While there is evidence that intense exercise can exacerbate arthritic symptoms in affected individuals (41, 42), physical therapy can significantly ease symtoms (43). These data have led to the idea of a “Goldilocks zone” in the joint (40), where the positive effects of movement are not yet overcome by the negative effects of the inflammation caused by joint loading.

In our studies herein, we identified enrichment of both regulatory and pro-inflammatory effector T cells within the gut-derived T cells of the PLNs, with the critical distinction between healthy mice and those with arthritic disease being the balance between the effector potential of those populations. The importance and sensitivity of the balance between opposing effector T cell subsets makes the selective blockade of pro-inflammatory T cell recruitment to the joint a particularly important axis to target in the development of therapeutics for SpA. Since both pro and anti-inflammatory gut-derived cells require access to the circulation in order to reach the joint, broad blockade of systemic trafficking is sub-optimal as a potential treatment paradigm in SpA. However, our finding that acute injury results in the influx of gut-derived Foxp3+ regulatory T cells suggests that the joint has the capability to engage in preferential recruitment of specific T cell subsets through the expression of specific chemokines or cell surface adhesion proteins. Therefore, discovery of the mechanisms recruitment of specific T cells subsets to the joint is an important avenue of research, as this would allow selective blockade of pathogenic pro-inflammatory T cell recruitment while minimizing the disruption to the homeostatic function of other T cell subsets in the joint.

Together, data shown here and the findings of others demonstrating the presence of gut-tropic immune cells in the joints of those with SpA (18, 23, 24) suggest a model in which trafficking of gut T cells is an important link that connects immunological processes at the gut and distal sites including the joint. We propose a model in which IELs are recruited to the gut in response to the detection of microbial products (31). These IELs would then gain effector function, both pro- and anti-inflammatory, in response to microbial antigen and as yet incompletely characterized local conditions. These cells then re-enter circulation and become a component of systemic immune surveillance. Following this, tonic inflammation at the joint, resulting from normal loading and unloading of the joint during day-to-day activities, results in the local production of chemoattractants and the influx of immune cells from circulation, some of which had previously gained pro-inflammatory effector function in microbiome dependent interactions. Therefore, depending on the conditions at the gut and the resultant priming of the circulating pool of potential respondent T cells, immune responses in the joint can either result in resolution or exacerbation of the initial inflammatory insult. Our data using broad spectrum antibiotics demonstrate that by removing microbial signals/antigens from the system, this link can be cut, and disease ameliorated. This is likely mediated by one of two mechanisms: Either the lack of IELs at the epithelium due to antibiotic treatment (31) results in the absence of some epithelial-cell dependent mechanism for licensing of IELs, or the lack of microbial antigen results in an absence of T cell priming in the mesenteric lymph node.

While we show gut-joint trafficking of IELs, here we do not address other potential means of disease transference between the gut and distal sites, such as the production of soluble factors at the gut that circulate and activate cells locally in the joint. Indeed, this mechanism may synergize with what we report here, as migratory IELs in the joint, which display heightened cytokine competence and enrichment for a Th17 phenotype, would be well positioned to respond to such soluble factors as IL-23 which are proposed to be produced in the gut in SpA (44). This study is also limited in that a targeted, specific deletion of IELs is not possible due to the heterogenous nature of IELs. Instead, we have chosen to employ a transfer strategy, which has the advantage of allowing the direct observation of the impact of IELs on responses in the joint but fails to account for the potential interactions between IELs and other cells of the adaptive immune system in the enthesial microenvironment.

In the TNF^ΔARE/+^ model used here, all TNF producing cell types are affected and consequently locally produced TNF as well as that derived from cells trafficking to sites of inflammation are important for the pathophysiology of disease. Indeed, TNF mRNA stabilization specifically in the colon epithelium is sufficient to drive gut pathology (45). However, results reported here demonstrate that broad-spectrum antibiotics delivered in the gut significantly reduces joint pathology and confirms the importance of cytokines derived from infiltrating cells for the development of inflammatory disease in the joint. Yet, our reported results showing that transfer of IELs from a TNF^ΔARE/+^ into a healthy recipient is insufficient to drive disease directly, but can serve to exacerbate existing inflammation, highlights the importance of the interaction between immune infiltrate and the local cellular milieu.

Altogether, our findings corroborate the general idea of the gut-joint hypothesis in SpA and showcase trafficking of IELs to the periphery as an important means by which this connection is mediated. Importantly, our finding that application of broad-spectrum antibiotic treatment resulted in a reduction of disease severity in the joint indicates a variety of druggable targets in the treatment of SpA. Further work will be required to determine the specific mechanisms by which IELs gain pro-inflammatory effector function, as well as how specific subsets are recruited the joint. As these targets would have a direct effect on the pathogenesis of disease and would not rely on blocking the deleterious effects of already in-progress pathogenic immune cascades, they would have the potential to more significantly limit disease progression.

## Data availability statement

The raw data supporting the conclusions of this article will be made available by the authors, without undue reservation.

## Ethics statement

The animal study was reviewed and approved by University of Colorado Anschutz Medical Campus Institutional Animal Care and Use Committee.

## Author contributions

AL and KK designed the study, executed experiments, analyzed data, and drafted the manuscript. EN, DC, and UK assisted with experimentation and data analysis. All authors approved the final version of the manuscript.

## Funding

The investigators and work presented here have been supported by NIH grants T32 AR007534 (ARL), K08 DK107905 (KAK), and R01 AR075033 (KAK) as well as funding from the Rheumatology Research Foundation K Supplement, Spondyloarthritis Research and Treatment Network, Spondylitis Association of America, Department of Medicine Outstanding Early Career Scholars Program, and the Shear Family Foundation (to KAK). Some work presented here was performed at the Barbara Davis Center Cell and Tissue Analysis Core at the University of Colorado Diabetes research Center with support from NIDDK P30 DK116073 and within the Mucosal Immunobiology of Rheumatologic Diseases Mucosal Inflammation Core P30 AR079369.

## Conflict of interest

The authors declare that the research was conducted in the absence of any commercial or financial relationships that could be construed as a potential conflict of interest.

## Publisher’s note

All claims expressed in this article are solely those of the authors and do not necessarily represent those of their affiliated organizations, or those of the publisher, the editors and the reviewers. Any product that may be evaluated in this article, or claim that may be made by its manufacturer, is not guaranteed or endorsed by the publisher.
