# Supplementary material for: Cytokine competent gut-joint migratory T Cells contribute to inflammation in the joint
Source: Front Immunol. 2022 Sep 7;13:932393. doi: 10.3389/fimmu.2022.932393 (PMC9489919; doi:10.3389/fimmu.2022.932393)
Supplement: Supplementary file 1 [file Table_1.docx]

**Supplementary Table 1:** **Antibodies used for spectral flow cytometry experiments**

| Target | Manufacturer | Clone | Flourophore |
| --- | --- | --- | --- |
| Viability | Tonbo | Ghost 510 | N/A |
| CD4 | Tonbo | RM4-5 | BV 450 |
| TCRβ | Tonbo | H57-597 | APC |
| CD8α | Tonbo | 53-6.7 | PE-Cy5 |
| CD8β | Biolegend | YTS156.7.7 | AF700 |
| CD69 | Invitrogen | H1.2F3 | PE-Dazzle 594 |
| CD103 | BD Bioscience | M290 | BUV 395 |
| TCRgd | Invitrogen | eBioGL3 | Percp-cy5.5 |
| CD44 | Biolegend | IM7 | BV 605 |
| CD62L | Biolegend | MEL-14 | APC-Cy7 |
| Foxp3 | Tonbo | 3G3 | PE-Cy7 |
| TNF | BD Bioscience | MP6-XT22 | BV711 |
| IL-17A | Biolegend | TC11-18H10.1 | BV605 |
| RorγT | BD Bioscience | Q31-378 | BV 786 |
