# Supplementary material for: Cytokine competent gut-joint migratory T Cells contribute to inflammation in the joint
Source: Front Immunol. 2022 Sep 7;13:932393. doi: 10.3389/fimmu.2022.932393 (PMC9489919; doi:10.3389/fimmu.2022.932393)
Supplement: Supplementary file 2 [file DataSheet_1.pdf]

Supplementary Figure 1

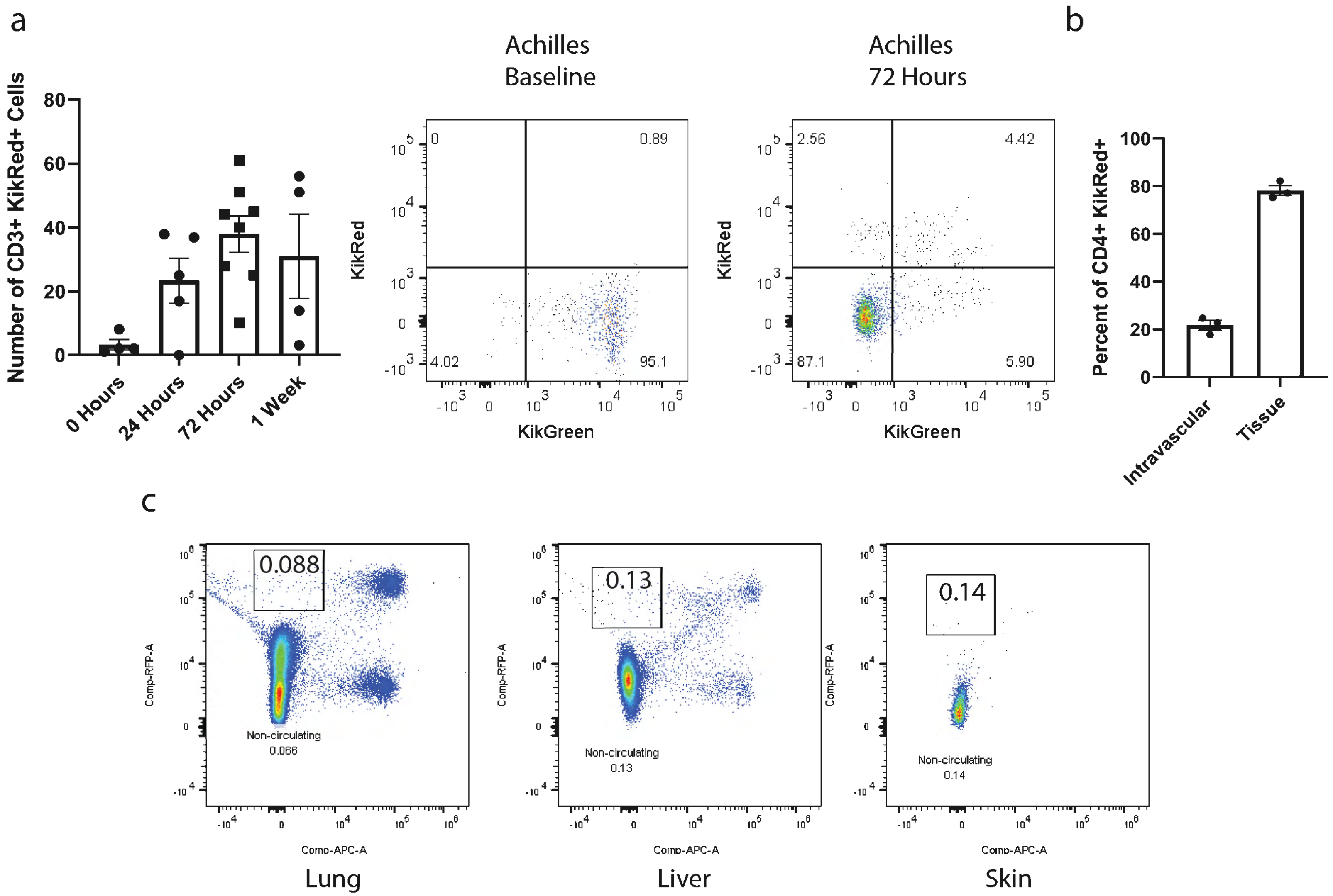

Supplementary Figure 1: IEL trafficking to the Achilles enthesis peaks at 72 hours and IEL trafficking to multiple target tissues

8-10 week old male and female KikGR+ mice underwent photoconversion of the distal colon and Achilles enthesis (a) was harvested at the indicated time. KikRed+ CD3+ T cells were enumerated by flow cytometry (left). Representative flow plots demonstrating KikGreen+ (x-axis) and KikRed+ (y-axis) CD3+ TCRαβ+ T cells before and after photoconversion (right). (b) Three 8 week old KikGR+ mice underwent photoconversion and were sacrificed 72 hours later. Four minutes before euthanasia, APC labeled anti-CD3 was inject IV to allow distinction of T cells in the circulation (APC+) versus T cells in the tissue (APC-). (c) An 8 week old male KikGR+ mouse underwent photoconversion of the distal colon and injection of APC labeled anti-CD3 as in (b) Lung, Liver, and Skin was collected and KikRed+ APC- cells enumerated by flow cytometry. Data are shown as individual animals across two separate experiments with bars as mean ± SEM.

Supplementary Figure 2

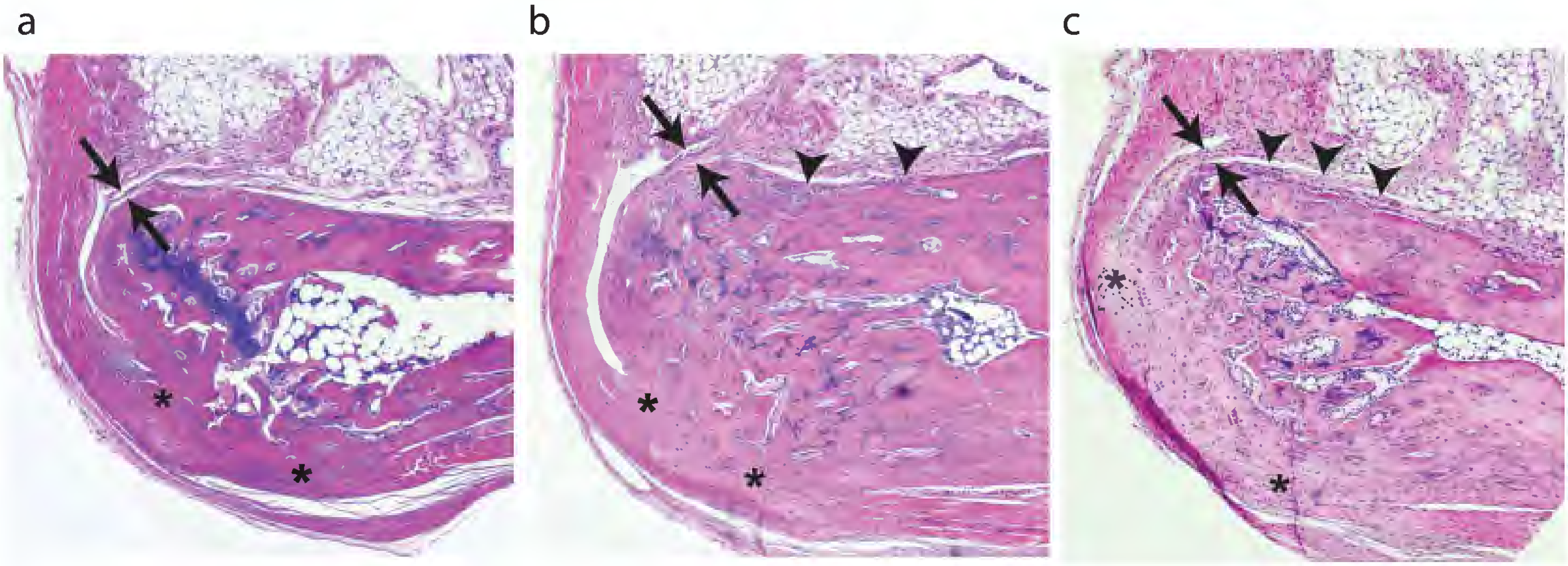

Supplementary Figure 2: Injection with 20mg CFA into the hind hock induces transient neutrophil infiltration, synovitis and enthesitis.
8-10 week old mice male and female KikGR+ mice were either sham injected with PBS (a) or received an injection of 20mg CFA in one hind hock (b, c). Animals were euthanized 3 days following administration of CFA and ankles evaluated histologically. Representative histology taken at 10x is shown. Arrows indicate the synovium, which is thickened in the CFA injected hocks. Arrowheads indicate periostial inflammation. Asterisks are the Achilles' and plantaris entheses.

Supplementary Figure 3

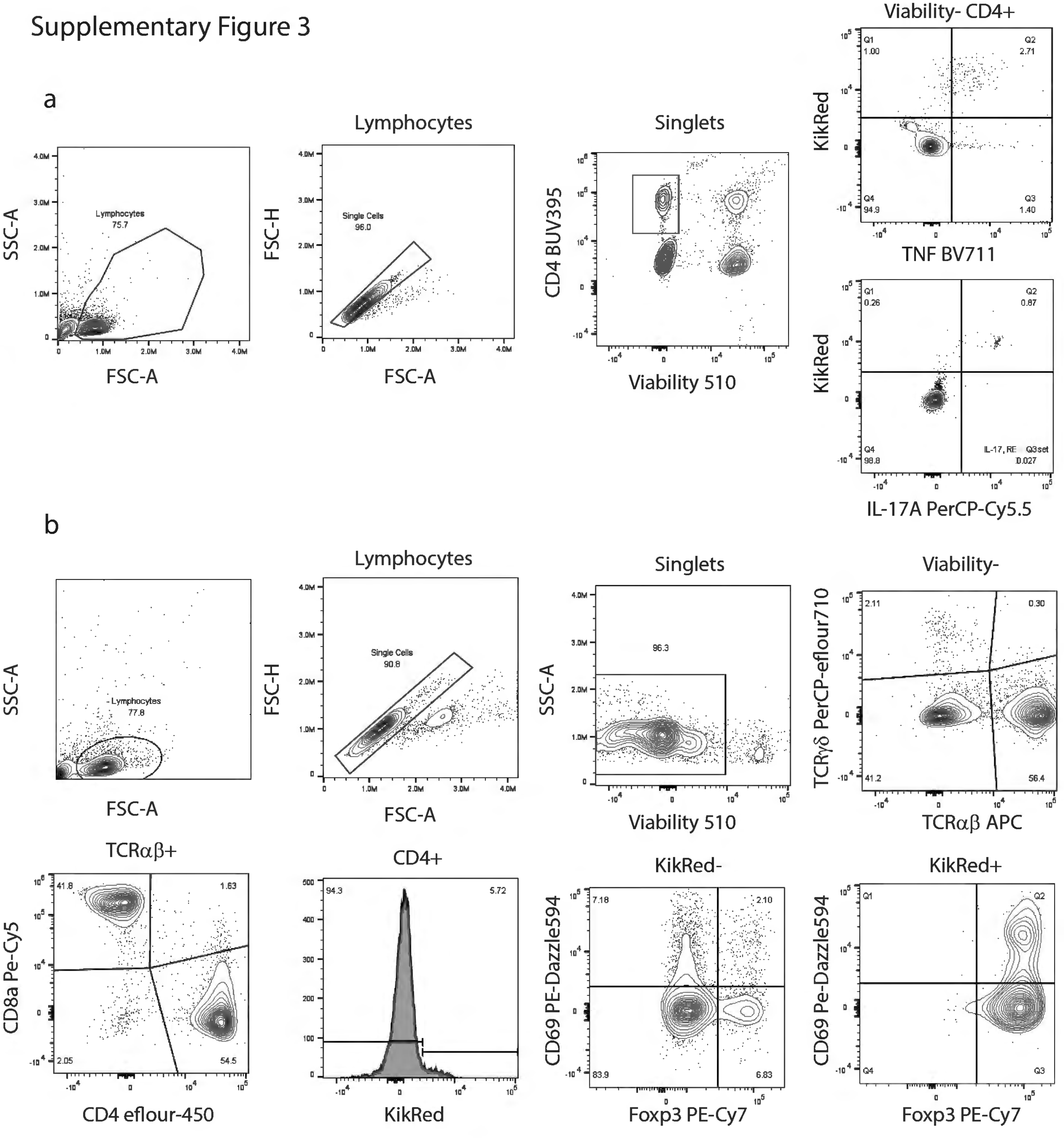

Supplementary Figure 3: Representative gating strategies for cytokines and T cell phenotypes.

Representative flow cytometry gating scheme for intracellular cytokine staining. (a) Size and doublet exclusion was applied before viable CD4+ T cells were identified and the cytokine expression of KikRed+ and KikRed- cells was determined. 20mg CFA was injected into one hind hock and both PLNs removed. Representative flow cytometry of the injection side is shown (b): size, doublet, and viability exclusion was applied before TCRαβ+, CD4+ cells were identified and Foxp3 and CD69 expression evaluated in the KikRed- (left) and KikRed+ (right) populations.

Supplementary Figure 4

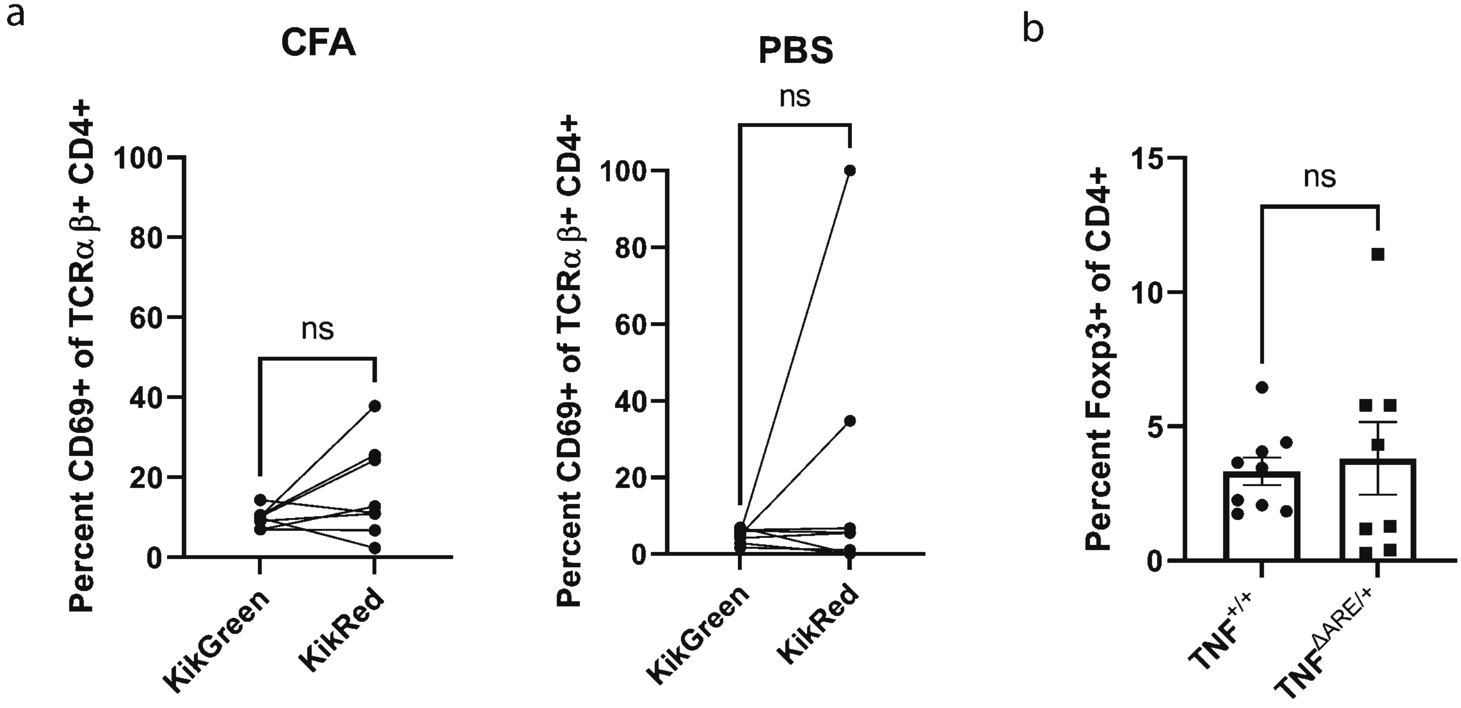

Supplementary Figure 4: CD69 expression and Foxp3+ T cell levels are similar in TNF$^{+/+}$ and TNF TNF$^{\Delta ARE/+}$ mice following hock injection.

(a) 8-10 week old male and female KikGR^{+} TNF$^{+/+}$ mice (n=7) received an injection of 20mg CFA in the hind hock and a control injection of PBS in the contralateral hock simultaneously to having undergone photoconversion of the distal colon. 72 hours post injection each Achilles enthesis was harvested and Foxp3+ TCRαβ+ CD4+ T cells were enumerated by flow cytometry. (b) 8-10 week old male and female KikGR^{+} TNF$^{\Delta ARE/+}$ (n=8) and TNF$^{+/+}$ littermate controls (n=9) received an injection of 20mg CFA in the hind hock and a sham injection of PBS in the contralateral hock simultaneously to having undergone photoconversion of the distal colon. 72 hours post injection each Achilles enthesis was harvested and Foxp3+ TCRαβ+ CD4+ T cells were enumerated by flow cytometry. Data are shown as individual animals across three separate experiments with bars as mean ± SEM. Significance determined by Student's t-test.

Supplementary Figure 5

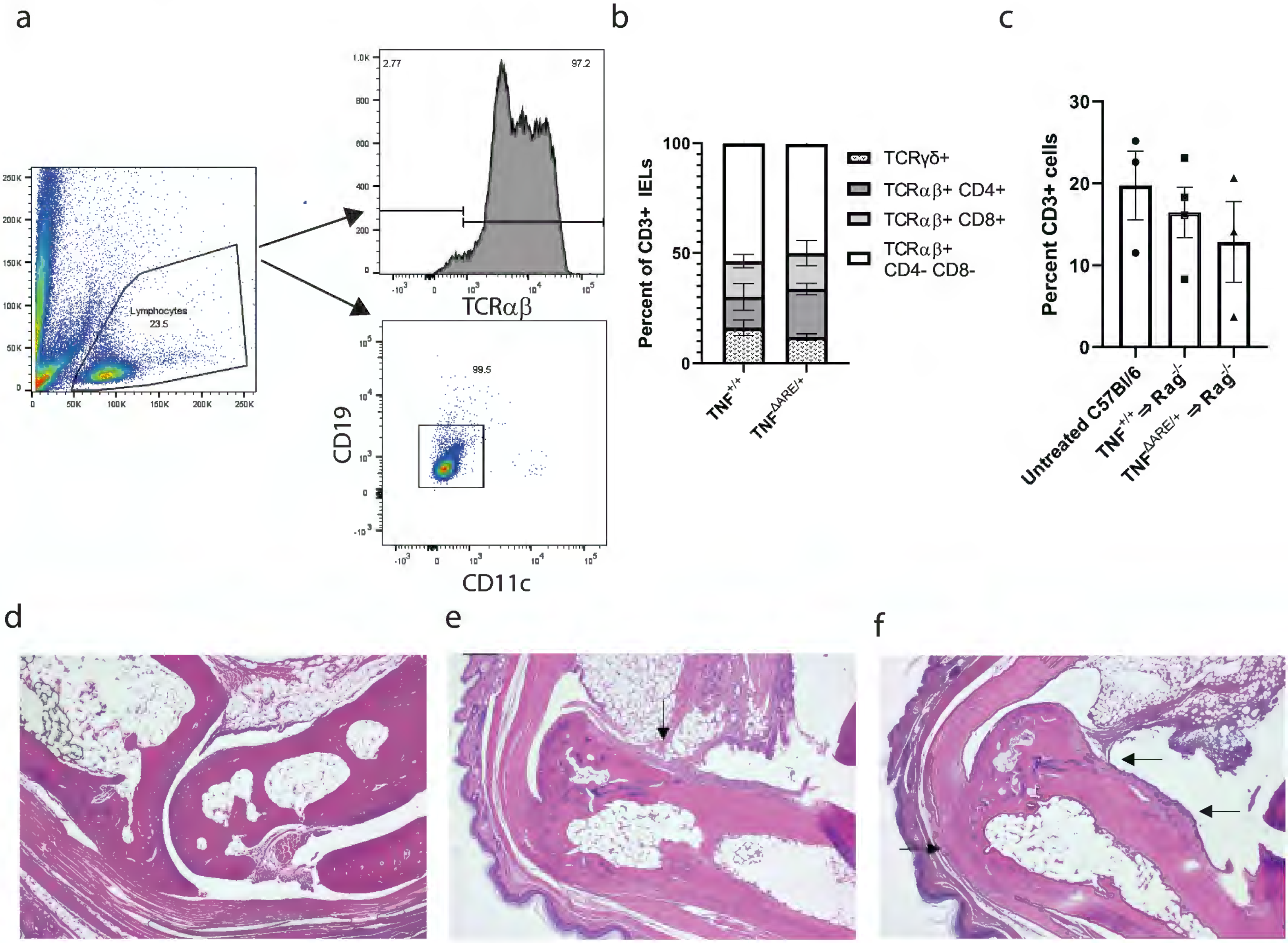

Supplementary Figure 5: Flow cytometry confirms ~99% purity of enriched IELs, and presence of circulating T cells three weeks post transfer.

IELs were harvested from the colon epithelia and enriched magnetically. (a) Post-enrichment purity was assessed by flow cytometry. (b) Comparison of IEL composition between 8 week old TNF$^{+/+}$ and TNF$^{\Delta ARE/+}$ littermates. (c) Peripheral blood was harvested by cheek bleed and circulating CD3+ T cell levels evaluated by flow cytometry. Following 10 weeks of homeostatic proliferation, RAG$^{-/-}$ controls (d), and recipients of IELs derived from TNF$^{+/+}$(e) or TNF$^{\Delta ARE/+}$ (f) received an injection of 20mg CFA in one hind hock. Animals were euthanized 5 days following administration of CFA and ankles evaluated histologically at 40x magnification.
